# Palmar-plantar erythrodysesthesia secondary to docetaxel chemotherapy: a case report

**DOI:** 10.1186/1752-1947-5-80

**Published:** 2011-02-25

**Authors:** Helen Benghiat, Amjad Al-Niaimi

**Affiliations:** 1 1Cancer Centre, University Hospital of North Staffordshire, Stoke-on-Trent, Newcastle Road, Staffordshire, ST4 6QG, UK

## Abstract

**Introduction:**

Docetaxel is a chemotherapeutic agent used alone or in combination for the management of many neoplastic conditions. Numerous side effects are well described as a consequence. Palmar-plantar erythrodysesthesia, although a relatively common side effect of some types of chemotherapy, occurs infrequently with docetaxel and is often attributed to other drug agents.

**Case Presentation:**

We report the case of a 66-year-old Caucasian woman who received adjuvant docetaxel monotherapy for invasive breast cancer. She developed palmar-plantar erythrodysesthesia following her first cycle of treatment, which necessitated a change in management.

**Conclusion:**

Palmar-plantar erythrodysesthesia is a relatively common side effect of cytotoxic chemotherapy, particularly with drugs such as 5-fluorouracil, capecitabine and liposomal doxorubicin. Docetaxel is commonly used both alone and in combination with a number of these agents for the management of various malignant conditions. We would like to highlight the occurrence of palmar-plantar erythrodysesthesia as a result of docetaxel monotherapy so that it can be considered as a potential cause in patients receiving combination treatment with chemotherapeutic agents better known to cause this toxicity.

## Introduction

Docetaxel is a frequently used chemotherapeutic agent belonging to the taxane family. It was first discovered in 1986 and is a semi-synthetic compound derived from the needles of the European yew (*Taxus baccata*) [1]. It exerts its anti-cancer effect by promoting microtubule stabilization, leading to mitotic arrest and subsequent cell death [2]. It is used alone or in combination with other chemotherapeutic agents for the management of various malignant conditions including breast, prostate, gastric, head and neck and non-small cell lung cancer [3]. Docetaxel is known to cause a number of side effects including hypersensitivity reactions, alopecia, nausea, vomiting, peripheral neuropathy, myelosuppression, diarrhea, mucositis, fluid retention, myalgia, arthralgia, nail changes and cutaneous reactions [3]. A number of skin reactions have been described in association with docetaxel, of which limb and/or palmar-plantar erythematous (PPE) reactions and fixed plaque erythrodysesthesia are amongst the more common [4].

## Case presentation

**Figure 1:**
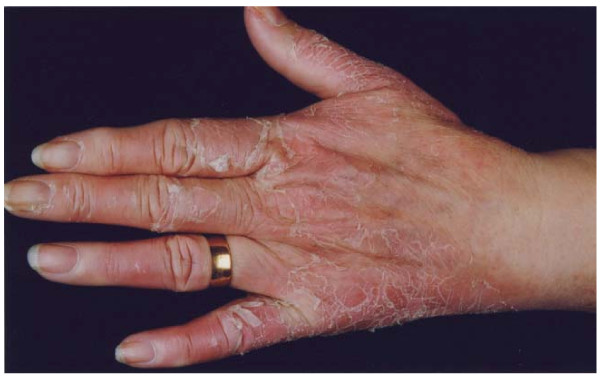
**Erythema with desquamation of the hand following docetaxel**.

**Figure 2:**
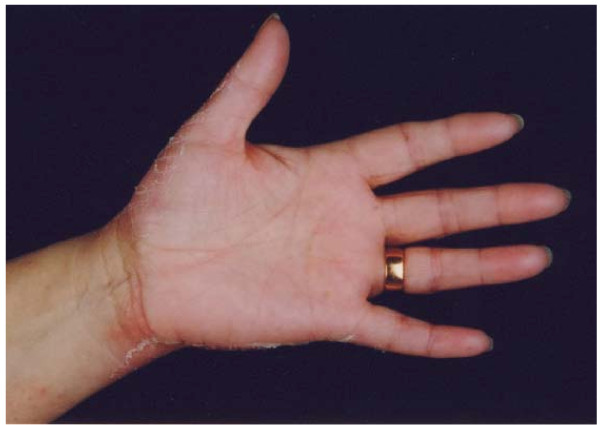
**Palmar erythema with desquamation following docetaxel**.

A 66-year-old Caucasian woman was referred to us for further investigation after noticing a lump in her right breast. Initial investigations were performed and she proceeded to have a right mastectomy and axillary node clearance. Histology revealed an 11.5 cm invasive lobular carcinoma with 8 out of 10 axillary lymph nodes containing metastatic disease. No distant metastases were evident after full staging investigations, hence her TNM stage was IIIA [5]. In accordance with local policy she was to be treated with three cycles of adjuvant 5-fluorouracil (500 mg/m^2^), epirubicin (100 mg/m^2^) and cyclophosphamide (500 mg/m^2^) (FEC) followed by three cycles of docetaxel (100 mg/m^2^) chemotherapy. She completed three cycles of FEC without toxicity and was commenced on docetaxel 21 days later as per protocol. Two days after receiving docetaxel she developed significant pain in her hands and feet. This was followed eight days later with marked erythema and swelling of both her hands, which interfered with her functional activities. The swelling subsided and marked skin desquamation occurred over the subsequent week (Figures 1 and 2). This reaction was classified as grade 3 PPE as defined by the National Cancer Institute grading system [6].

In agreement with the patient, the docetaxel chemotherapy was discontinued and regular emollients were prescribed. The patient was restarted on FEC chemotherapy and received a further three cycles without toxicity. One month after the docetaxel, her hands had significantly improved, with only mild erythema persisting.

## Discussion

PPE is a relatively common side effect of many types of chemotherapy, namely 5-fluorouracil, capecitabine and liposomal doxorubicin [7]. It has been reported in association with docetaxel in a number of case reports, initially by Zimmerman *et al. *in 1994 [8], but remains rare. PPE initially presents with erythema and dysesthesia in the palms and soles. This may progress to pain with dryness, desquamation and ulceration. Although a number of classification systems exist, many UK based cancer centers grade PPE as per the National Cancer Institute. This defines grade 1 toxicity as skin changes or dermatitis without pain, grade 2 as skin changes with pain not interfering with function, and grade 3 toxicity as skin changes with pain interfering with function [6].

The etiology of PPE, and why it occurs more frequently with some chemotherapeutic drugs, remains unclear and as such treatment remains symptomatic. Currently, the most effective management is treatment interruption and dose reduction [9]. Some success has been achieved with the use of emollients and a number of small studies and anecdotal reports have shown oral pyridoxine to be useful both as a preventative and therapeutic measure [7].

## Conclusion

PPE is a recognized toxicity occurring most commonly with 5-fluorouracil, capecitabine and liposomal doxorubicin chemotherapy. Docetaxel is frequently used in combination with 5-fluorouracil and capecitabine for the management of various malignant conditions including breast, head and neck and gastric cancer [3]. When PPE occurs as a result of the use of these combination regimens, although not as commonly implicated, docetaxel should be considered as a potential cause to avoid mismanagement.

## Consent

Written informed consent was obtained from the patient for publication of this case report and any accompanying images. A copy of the written consent is available for review by the Editor-in-Chief of this journal.

## Competing interests

The authors declare that they have no competing interests.

## Authors' contributions

HB and AAN both managed the case. HB wrote the manuscript. Both authors read and approved the final manuscript.
